# Quantum correlated heat engine in *XY* chain with Dzyaloshinskii–Moriya interactions

**DOI:** 10.1038/s41598-022-11146-3

**Published:** 2022-04-30

**Authors:** M. Asadian, S. Ahadpour, F. Mirmasoudi

**Affiliations:** Department of Physics, University of Mohaghegh Ardabili, 56199-11367 Ardabil, Iran

**Keywords:** Condensed-matter physics, Quantum physics, Statistical physics, thermodynamics and nonlinear dynamics

## Abstract

In this paper, we consider a heat engines composed of two interactional qubits with spin-orbit interaction (Dzyaloshinskii–Moriya (*DM*)) subject to an external magnetic field, so that each qubit is coupled with cold or hot source. One intention of this work is to investigate the following question: is it possible the effects of *DM* lead to improve basic thermodynamic quantities in this heat engine are coupled to local environments that are not necessarily at equilibrium? Moreover, we study whether or not quantum correlations can be helpful in the performance of quantum work engines. For this end, we investigate the effects of the temperature and the interaction rate of each qubit with its surrounding environment on quantum correlations such as quantum coherence and quantum discord and quantum entanglements, as well as the generated work. Finally we compare three quantum correlations (entanglement, discord, and coherence) with thermodynamic parameters and show that the output work is positive for what values of the magnetic field so that this cycle can be considered as a thermal machine.

## Introduction

In recent years, there has been a revolution in the thermodynamic concepts, which is referred to as a new found among the physical theories. In this theory, the spread of information is described through quantum systems. This new theory, under the title of quantum thermodynamics, attempts to express thermodynamic concepts such as work, heat, efficiency, and so on by using quantum information theory^1–4^. A primary relation exists between thermodynamics and information theory. The laws of thermodynamics are a set of universal rules that explain the interaction between temperature, heat, work, energy, and entropy. The laws of thermodynamics are not only valid in the case of steam engines, but also for any other problem, including the sun, black holes, living things, and even the whole world. Thermodynamic rules are used only to express that a process is feasible or not. Quantum thermodynamics works to develop quantum machines^5–8^. The most important application of quantum thermodynamics is quantum heat engines and one of the famous cycles in the quantum thermodynamic is the Carnot cycle^9–11^. The most common cyclic process is the Otto cycle^12–14^. The Otto cycle is the basis of all the heat engines performed based on the internal combustion process and hence it is of critical importance. In the following, we will begin to describe the basic processes (strokes) related to the classical form of the Otto cycle, in which the active material is a two-level system. The name of Otto cycle is retrieved from the German engineer Nicholas Otto, who worked for the manufacturing of the first four-stroke engine based on the initial design presented by *Alphonse* *Beau* *de* *Rochas*. Here, we want to discuss the role of quantum correlations in quantum thermodynamics^15^. First, *Alicki* defined the weak connection between heat and work in thermodynamic processes, namely under the influence of slowly varying external conditions. He assumed that a change in the Hamiltonian and state of the sub-system is necessarily associated with the work and heat, respectively^16^. Afterward, more appropriate definitions are presented for thermodynamic work and heat^17–20^. Different quantum matters such as qubits^21–25^, qudits^26–29^, photons^30–32^, harmonic oscillators^13,33,34^, and so on are used in the field of heat engines. In this paper, a system is considered with two qubits *a* and *b* as the working matter. We consider an Otto cycle with the working matter in the form of two coupled qubits. We compare the behavior of quantum correlation and thermodynamic parameters together. This model we have considered show that there is a connection between quantum correlations and positive work in cycle.

The paper is organized as follows. In "Model system: the quantum Otto engine" Section, we give the model and calculate the density matrix for a system composed of two interactional qubits with spin-orbit interaction in the Otto cycle. In "Work definition" Section, we define work and calculating the work, heat and efficiency of a heat cycle for our proposed model. In "Quantum correlations" Section, we recall briefly entanglement, discord and coherence measures of the quantum correlations and we derive the analytical expression to the entanglement, discord and coherence and show how creating quantum correlations can be limited by the thermodynamics of the system. Finally, we give the conclusions in "Conclusion" Section.

## Model system: the quantum Otto engine

Here we consider a system consisting of two interacting qubits *a* and *b* as the working material a four- level quantum Otto engine, which is described by *XY* Hamiltonian considering the spin-orbit interaction and magnetic field^35^:

1 $$\begin{aligned} H = (J_x \sigma _{x_a} \sigma _{x_b} + J_y \sigma _{y_a} \sigma _{y_b}) + B(\sigma _{z_a} + \sigma _{z_b}) + D(\sigma _{x_a} \sigma _{y_b} - \sigma _{y_a} \sigma _{x_b}) \end{aligned}$$

where $\sigma _{[x, y, z]_j}$ denotes the Pauli operators that affect the qubits $j = a, b$, $J_x$ and $J_y$ are the strength of the antiferromagnetic couplings, *B* is the intensity of the magnetic field, and *D* is the spin-orbit interaction factor. We set $J_x= J_y=J$ and assume that the dynamics of the working matter density operator $\rho$ have the Markovian property, and can be stated by *Lindblad* master equation as follows^36^:

2 $$\begin{aligned} {\dot{\rho }} = -[H,\rho ] + \sum _i g_i L_{a_i} (\rho ). \end{aligned}$$

Accordingly, $L_{a_i} = 2a_i \rho _{a_i}^+ - \{a^+_i a_i, \rho \}$ and $a_i$ is the jump operator that describes the operation of the baths, and $g_i$ is the dissipation rate associated with the *Lindblad* term $L_i$ in the above equation. It is assumed that *Lindblad* operators of $L^j_{\sigma _+}$ with coefficient $g^j_+=\gamma _j{{\overline{n}}}_j$, and $L^j_{\sigma _-}$ with coefficient $g^j_-=\gamma _j({{\overline{n}}}_j+1)$ are the jump operators in two general forms of raising operator $\sigma _+=| 1\rangle \langle 0|$ and lowering operator $\sigma _-=| 0\rangle \langle 1|$. The coefficients $\gamma _j$ is the interaction rate of each qubit with its surroundings and ${\overline{n}}_j$ is the population of the corresponding equilibrium temperature (the average particle number) which is defined by the following equation:

3 $$\begin{aligned} {\bar{n}}_j = (e^{2B_j/T_j} -1)^{-1} \end{aligned}$$

where $B_j$ and $T_j$ are the magnetic field and temperature for the bath coupled to qubit *j*, respectively. In the present paper, we consider different temperatures for the cold and hot baths coupled with two qubits. So, ${\overline{n}}_{C_a}$ and ${\overline{n}}_{C_b}$ are the population of the corresponding equilibrium temperature of the cold bath coupled with qubits *a* and *b* in the cooling process, and ${\overline{n}}_{H_a}$ and ${\overline{n}}_{H_b}$ are the population of the corresponding equilibrium temperature of the hot bath coupled with qubits *a* and *b* in the heating process. It is assumed that $\gamma _a=\gamma _1$ and $\gamma _b=\gamma _2$ (breakdown rates) take on different values. In this generalized Otto cycle, during the compression (expansion) stroke the magnetic field and spin-orbit interaction factor are changed, for both qubits, from $B_1$ to $B_2$ and $D_1$ to $D_2$ ($B_2$ to $B_1$ and $D_2$ to $D_1$) with $\mathrm {B_2}>\mathrm {B_1}$ and $\mathrm {D_2}>\mathrm {D_1}$. Under this condition, by following Ref^36^ the density matrix can be expressed as,

4 $$\begin{aligned} {\rho _s} = \dfrac{1}{\alpha } \begin{pmatrix} r_{11} &{} 0 &{} 0 &{} 0 \\ 0 &{} r_{22} &{} ir_{23} &{} 0 \\ 0 &{} -ir_{23} &{} r_{33} &{} 0 \\ 0 &{} 0 &{} 0 &{} r_{44} \end{pmatrix} \end{aligned}$$

where *r*’s and $\alpha$ are reported in Appendix. The working substance undergoes a generalized Otto cycle^37^. The four stages of the cycle are as follows:

1. Adiabatic compression (Isentropic): The system is compressed, and work is done on the system. This stroke consists of volume and temperature variations, while entropy will remain constant throughout it. The material is isolated from the environment, and some of the Hamiltonian parameters $J_x$, $J_y$ , *B* or *D* changes accordingly to involve an increase in the energy gap.
2. Isochoric heating: The temperature increases while the volume of the system (working substance) is constant. That means heat is absorbed from the source. During the evolution, the working matter proceeds to a steady state of Equation (2) with ${\dot{\rho }} = 0$.
3. Adiabatic expansion (Isentropic): The stroke power when a useful work comes out of the engine. Again, this stroke involves volume and temperature variations, and entropy is constant. The material is isolated from the environment, and some of the Hamiltonian parameters $J_x$, $J_y$ , *B* or *D* gets its previous value. This leads to a decrease in the energy gap, which means approaching the classic case.
4. Isochoric cooling: The environment is cooled in a constant volume and returns to its initial state, and it is ready to start the cycle again. To understand all aspects of the Otto-quantum cycle, we should examine how this four-stroke cycle is performed in the work environment which is a quantum system.

We note that for Hamiltonian Eq. (1) the parameter *P*(*t*) changes depending on the evolution of each of the parameters $J_x$ , $J_y$, *B* or *D*. This parameter revolves from the initial $P_1$ to the final value $P_2$ through the equation $P(t)=P_1+(P_2-P_1)t/\tau$, where $\tau$ is the duration of the work stroke. In the following, we will study the quantum work and three quantum correlations (entanglement, coherence, and discord) for this density matrix and compare the behavior of these three quantum correlations. We also present the variation of the work against different parameters.

## Work definition

In classical thermodynamics, we can define work as the potential energy of an external device that can be stored for later uses. In quantum systems, work is defined as a change in the energy of two systems^17^. To assess the work extracted or produced by the engine during the cycle we employ the definition of work based on the two-time measurement protocol. We now change the Hamiltonian from $$ H_{in}$$ to $$H_{fin}$$ in time while the system is isolated from any environment. $$ H_{in} $$ and $$H_{fin}$$ are the initial Hamiltonian with eigenvalues $$E_i^{in}$$ and the final Hamiltonian with eigenvalues $$E_i^{fin}$$, respectively. Assuming $$\rho _\tau =U {\rho _s} U^+$$ we can obtain final state, in this equation $$\rho _s$$ is the initial state of the system in the process and $$\rho _\tau $$ is the final state of the process where *U* is a unitary operator. Now, the mean work can be obtained as:

5 $$\begin{aligned} W= \mathrm {Tr}(\rho _\tau {H_{fin}})-\mathrm {Tr}(\rho _s{H_{in}}), \end{aligned}$$

An important property in the Otto cycle is that it is isolated during the expansion and compression processes, and the change of energy is only in the form of external work. On the other hand, both work and heat variation may occur in cooling and heating processes and for unbalanced reservoirs. $\mathrm {-W_1}$ and $\mathrm {-W_2}$ represent the extracted work during the expansion and compression processes, and $\mathrm {Q_1}$ and $\mathrm {Q_2}$ are the heat obtained by the environment during heating and cooling processes. We assume $\mathrm {Q>0}$ when the heat is absorbed from the environment and the energy level increases in the system. Heat fluctuations cause the system to excite and the system leaves the ground state. Which means an increase in energy levels. So, we have for the cyclic evolution:

6 $$\begin{aligned} W_1+W_2+Q_1+Q_2=0. \end{aligned}$$

And the total derived work is equal to:

7 $$\begin{aligned} W_T=-(W_1+W_2)=Q_1+Q_2. \end{aligned}$$

In this case, efficiency is defined as the ratio of the extracted work (if positive) to the total heat absorbed by the source^15^:

8 $$\begin{aligned} \eta =\frac{W_T}{\sum _{Q_i>0}Q_i}. \end{aligned}$$

Assuming that the heating process corresponds to connecting the two qubits with two local baths with thermal occupation ${\bar{n}}_H$ and the cooling process with ${\bar{n}}_C$, on condition ${\bar{n}}_b = 0$, the following equations are obtained. Now, we assume $\rho ^C$ and $\rho ^H$ are the stable states for cold and hot baths with $r_{ij}^C$ and $r_{ij}^H$ elements, respectively. The total work is given by the following equation:

9 $$\begin{aligned} W_T = (B_2 - B_1) (r^C_{11} - r_{11}^H + r_{44}^H - r_{44}^C). \end{aligned}$$

We remark here that heat absorbed is minus the energy balance of the system during the heating of the system from ${\bar{n}}_C$ to ${\bar{n}}_H$. Thus, for the heat absorbed from two hot sources, using Eq. (7), we have:

10 $$\begin{aligned} Q_1 = B_2 (r^C_{11} - r^H_{11} + r_{44}^H - r_{44}^C). \end{aligned}$$

And the efficiency is defined as follows:

11 $$\begin{aligned} \eta = \dfrac{W_T}{Q_1} = 1 - \dfrac{B_1}{B_2}. \end{aligned}$$

**Figure 1:**
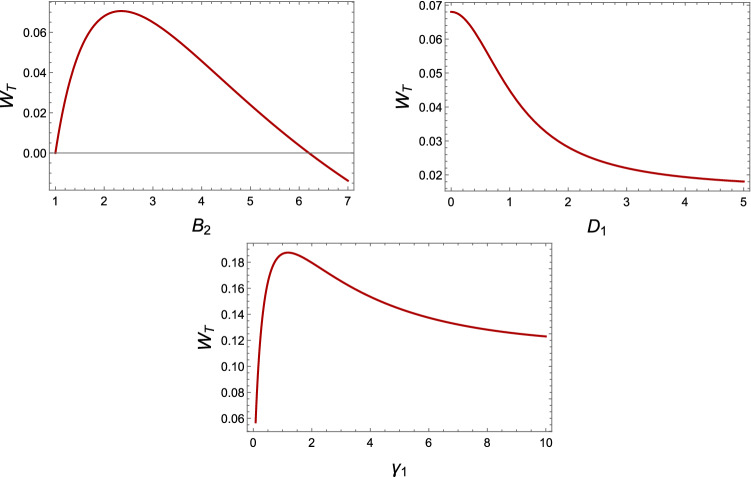
Plots of the total work for an Otto cycle operating between $T_1 = 1$ and $T_2 = 4$ using the steady state. (Top left): We fix $J = 1$, $D_1=0$, $D_2=5$, $\gamma _1=0.1$, $\gamma _2=5$, ${\bar{n}}_b=0$ and vary the magnetic field from $B_1= 1$ to $B_2$ reported on the horizontal axis. (Top right): We fix $J = 1$, $\gamma _1=0.1$, $\gamma _2=5$, $D_2=5$, $B_1 = 1$ and $B_2 = 2$; and vary the $D_1$ from 0 to 5. (Bottom): We fix $J = 1$, $B_1 = 1$, $B_2=2$, $D_1=0$, $D_2$=5, $\gamma _2 = 5$ ,${\overline{n}}_b=0$ and we change interaction rate of first qubit with its surrounding ($\gamma _1$) from 0 to the value reported on the horizontal axis.

**Figure 2:**
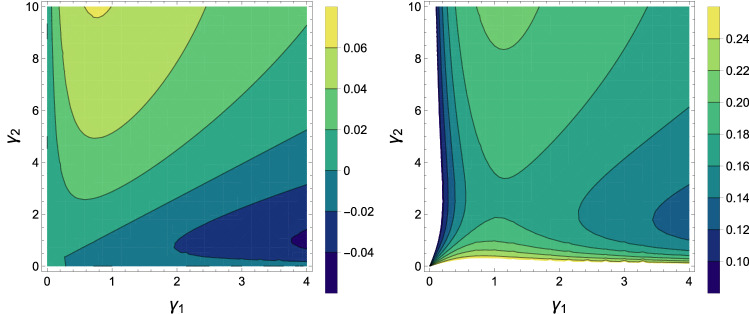
Contour plots of the total work for an Otto cycle corresponding to $T_2=2$ (left) and $T_2=4$ (right) as a function of $\gamma _1$ and $\gamma _2$, with $J = 1$, $B_1=1$, $B_2=2$, $T_1=1$, ${\bar{n}}_b = 0$, $D_1=0$ and $D_2=5$ for both panels.

It is straightforward to see that the efficiency only is under the effect of the magnetic field is identical on both spins can be controlled by it. But, the external work and heat not only depend on the magnetic field but also Dzyaloshinskii–Moriya. How one can detect the adiabatic process in this system? For the illustrating purpose, we will assume two different outlines for the adiabatic stages: At first, the Dzyaloshinski-Moriya (DM) anisotropic antisymmrtric interaction is altered between two values ($D_1\rightarrow D_2 \rightarrow D_1$ ) at a magnetic field, and next the fixed magnetic field is changed between two values ($B_1 \rightarrow B_2 \rightarrow B_1$ ) at a Dzyaloshinski-Moriya (DM) anisotropic antisymmrtric interaction. We calculate the total work for different parameters such as the interaction rate of the first qubit with its environment and the interaction factor between the spin-orbit and magnetic field. In Fig. 1, there is work for $\gamma _1\ge 0$ and it is maximum at $1\le \gamma _1 \le 2$. In Fig. 1, the output work is positive for $1\le B_2 \le 6$, so this idea can be implemented as a practical heat engine. Also we observed that the derived total work decreases by increasing the spin-orbit interaction factor. In Fig. 2 contour plots of the total work is plotted as a function $\gamma _1$ and $\gamma _2$. It is clear that for $T_2=4$ for $\gamma _1\approx 1$ and $\gamma _2\approx 0.2$ the total work have the maximum possible value. Also, we observe that by increasing the temperature of hot bath from $T_2=2$ to $T_2=4$, the total work is increased. Considering the Dzyaloshinskii–Moriya interactions, despite the increase in the population of the corresponding equilibrium temperature, the total work is still there. From Eq. (11), we see that increasing the magnetic field has a very positive effect on system efficiency. But increasing the temperature has no effect on efficiency, efficiency is sensitive to changes in magnetic field. We remark here that the two-time energy measurement protocol may affect both the work produced and the quantum correlations of the working substance, which is the main focus of this paper.

## Quantum correlations

In this section, we investigate the effects of quantum correlations at the end of the two isochoric processes of the Otto cycle. We discuss the quantum correlations in our system by analyzing the behavior of the entanglement, quantum discord and quantum coherence. Since, the elements of our density matrix are independent of the magnetic field, the quantum correlations will not be dependent on the magnetic field. Depending on the inter qubit couplings, the reservoir temperatures, and the decay rates, the steady state might become entangled. To measure the amount of the latter we employ the concurrence.

### Quantum entanglement

Entanglement is one of the important and Unintuitive phenomena in the quantum world. The entanglement is the property shared between two or more systems showing correlations that cannot be described by classical physics, and this kind of correlation does not exist in the macroscopic world. Two particles may be very far from each other, but they can relate to each other, and whatever happens to one, it immediately causes a change in the other one. In quantum mechanics, the entanglement relates to quantum correlations that are rooted in the inseparable nature of the state vector of the quantum system^15,38–40^. If $\rho$ is the density matrix of two qubits, so $\lambda _i$ represent the roots of matrix $\rho {\tilde{\rho }}$ where ${\tilde{\rho }}$ is defined as ${\tilde{\rho }}=(\sigma _{y_a}\otimes \sigma _{y_b})\rho ^*(\sigma _{y_a}\otimes \sigma _{y_b})$. Also, the concurrence as an entanglement measure, can be calculated with $C(\rho )=\max (0,\sqrt{\lambda _1}-\sqrt{\lambda _2}-\sqrt{\lambda _3}-\sqrt{\lambda _4})$, where $\lambda _1>\lambda _2>\lambda _3>\lambda _4$. Because our matrix is X-state, concurrence is obtained as follows:

12 $$\begin{aligned} C(\rho ) = \max [0, t_1, t_2], \end{aligned}$$

where $$t_1 = 2(\sqrt{r_{23} \times r_{32}} - \sqrt{r_{11} \times r_{44}})$$ and $$t_2= 2(\sqrt{r_{14} \times r_{41}} - \sqrt{r_{22} \times r_{33}})$$.

**Figure 3:**
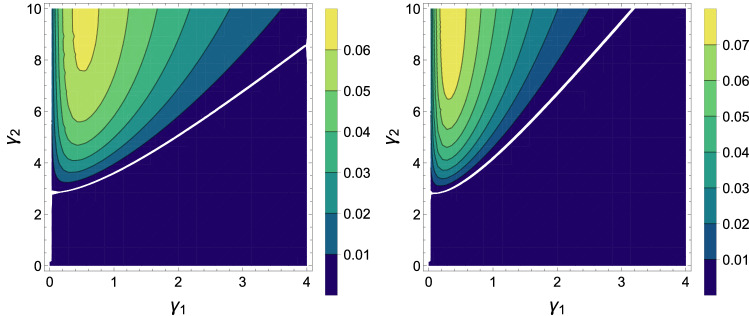
Contour plots of the concurrence of state $\rho$ in Eq. (4) corresponding to ${\bar{n}}_b = 0$ and ${\bar{n}}_a = 1$ (left) and ${\bar{n}}_a = 2$ (right) as a function of $\gamma _1$ and $\gamma _2$, with $J = 1$ and $D=1$ for both panels.

In Fig. 3 contour plots of the concurrence obtained from Eq. (12) is plotted as a function $\gamma _1$ and $\gamma _2$. We observe that the entanglement gets smaller by increasing the temperature. Even, the maximum amount of entanglement in this case is a small amount. We can see in Fig. 3 that the entanglement persists only for very low values of ${\bar{n}}$.

### Quantum discord

A bipartite quantum state consists of classical and conventional correlations. These correlations are jointly measured by their quantum mutual information which is a theoretical measure of the total correlation in a bipartite quantum state. In particular, if $\rho _{AB}$ is the density operator of a compound binary system *AB* and $\rho _A(\rho _B)$ describes the density operator of term *A*(*B*), thus the quantum mutual information is defined as^40^,

13 $$\begin{aligned} L(\rho (AB))=S(\rho _A)+S(\rho _B)-S(\rho _{AB}) \end{aligned}$$

where $S(\rho )=-\mathrm {Tr}(\rho \log _2{\rho })$ is the von *Neumann* entropy. Quantum mutual information may be written as the sum of the classical correlation $C(\rho _{AB})$ and the quantum correlation $Q(\rho _{AB})$ that is $L(\rho (AB))=C(\rho _{AB})+Q(\rho _{AB})$^41–44^. This quantum term $Q(\rho )$ is called quantum discord^42^. This is a different kind of quantum correlation compared to the entanglement since the separable mixed modes (i.e., without entanglement) can have a nonzero quantum discord. Quantum discord is not always bigger than the entanglement^45,46^. The quantum discord (*QD*) for the X-state matrix is obtained as follows^43^:

14 $$\begin{aligned} QD (\rho _{AB}) = \min (Q_1, Q_2), \end{aligned}$$

that $Q_1$ and $Q_2$ are obtained as follows,

15a $$\begin{aligned} \epsilon _1&= \dfrac{1}{2} \left[ (r_{11} + r_{44}) + \sqrt{(r_{11} - r_{44})^2 + 4|r_{14}|^2}\right] , \end{aligned}$$

15b $$\begin{aligned} \epsilon _2&= \dfrac{1}{2} \left[ (r_{11} + r_{44}) - \sqrt{(r_{11} - r_{44})^2 + 4|r_{14}|^2}\right] , \end{aligned}$$

15c $$\begin{aligned} \epsilon _3&= \dfrac{1}{2} \left[ (r_{11} + r_{33}) + \sqrt{(r_{22} - r_{33})^2 + 4|r_{23}|^2}\right] , \end{aligned}$$

15d $$\begin{aligned} \epsilon _4&= \dfrac{1}{2} \left[ (r_{22} + r_{33}) - \sqrt{(r_{22} - r_{33})^2 + 4|r_{23}|^2}\right] , \end{aligned}$$

16 $$\begin{aligned} H(x) = -x \log _2{x} - (1 - x) \log _2{(1 - x)}, \end{aligned}$$

17a $$\begin{aligned} d_1&= H \left( \dfrac{1 + \sqrt{[1 - 2(r_{33} + r_{44}]^2 + 4(|r_{14}| + |r_{23}|)^2}}{2}\right) , \end{aligned}$$

17b $$\begin{aligned} d_2&=\sum _i r_{ii} \log _2{r_{ii}} - H (r_{11} + r_{33}), \end{aligned}$$

18 $$\begin{aligned} Q_j = H(r_{11} + r_{33}) + \sum _{i=1}^4 \epsilon _i \log _2 {\epsilon _i} + d_j. \end{aligned}$$

**Figure 4:**
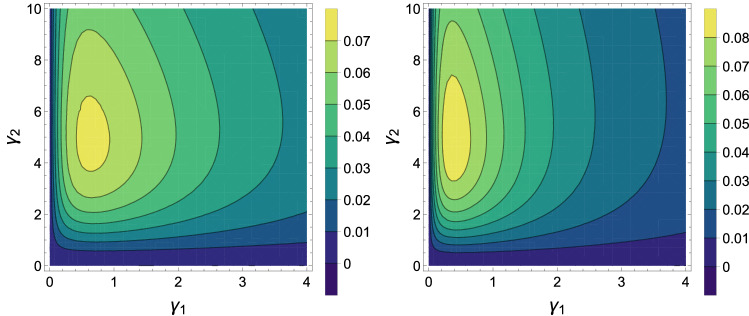
Contour plots of the discord of state $\rho$ in Eq. ( 4) corresponding to ${\bar{n}}_b = 0$ and ${\bar{n}}_a = 1$ (left) and ${\bar{n}}_a = 2$ (right) as a function of $\gamma _1$ and $\gamma _2$, with $J = 1$ and $D=1$ for both panels.

In Fig. 4 using the Eq. (14) contour plots of the quantum discord is plotted as a function $\gamma _1$ and $\gamma _2$. We find from Fig. 4 which quantum discord is sensitive to reservoir temperatures. It is important to note under what the condition quantum discord can be sustain more. Moreover, by compareing Figs. 3 and 4 we can find with increasing the interaction rate of the second qubit with its environment, the entanglement becomes more tangible and the quantum discord first increases, then decreases. Also, with increasing the population of the corresponding equilibrium temperature, both entanglement and quantum discord are decreasing.

**Figure 5:**
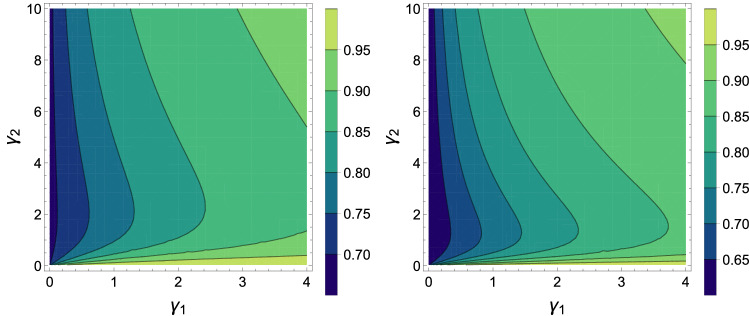
Contour plots of the coherence of state $\rho$ in Eq. (4) corresponding to ${\bar{n}}_b = 0$ and ${\bar{n}}_a = 1$ (left) and ${\bar{n}}_a = 2$ (right) as a function of $\gamma _1$ and $\gamma _2$, with $J = 1$ and $D=1$ for both panels.

### Quantum coherence

The quantum coherence derived from the superposition of quantum states differentiates quantum from classical mechanics. The quantum coherence contained between different parts of quantum systems is the basis for the creation of quantum correlations^47–49^. The quantum coherence, which arises from the superposition principle, is one of the best non-classical features of a quantum system. Based on the research works conducted in this field^50–52^, quantum coherence is achieved through the following equation:

19 $$\begin{aligned} I(\rho ,k)=-\frac{1}{2}\mathrm {Tr}[\sqrt{\rho },k]^2 \end{aligned}$$

where $\rho$ is the quantum state density matrix, *k* is an observable, and $[\; , \;]$ is the displacement. *Minimum* *skew* information achievable on a single local measurement defined as,

20 $$\begin{aligned} u_A \equiv min_{K^A}I(\rho _{AB}, K^A) \end{aligned}$$

The minimization is performed over all local maximally informative observable (or nondegenerate) $K^A = K_A \otimes I_B$. We use a closed form of the quantum coherence for $2 \otimes d$ quantum systems which is introduced by *Girolami* as,

21 $$\begin{aligned} u_A = 1 - \lambda _{\max } (\chi ) \end{aligned}$$

where $\lambda _{\max }$ is the maximum eigenvalue of the $3 \times 3$ matrix $\chi$ with the elements $\chi _{ij} = \mathrm {Tr}\{\sqrt{\rho } (\sigma _{i_A} \otimes I_{B}) \sqrt{\rho } (\sigma _{j_A} \otimes I_B)\}$ and $\sigma _i(i = 1, 2, 3)$ represent the Pauli matrices.

**Figure 6:**
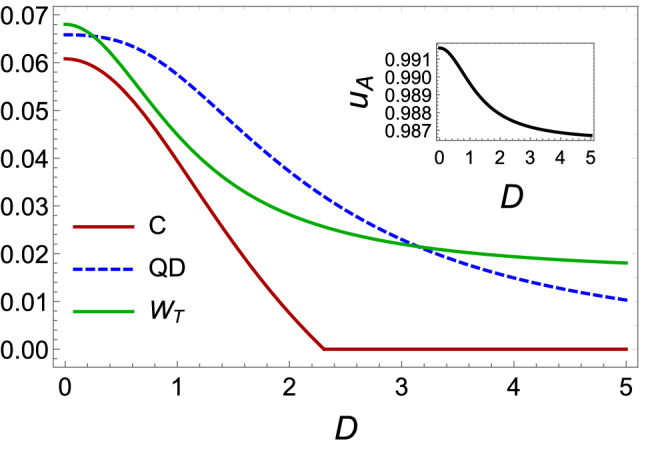
Plots of the total work, coherence, concurrence and discord for an Otto cycle operating between $T_1 = 1$ and $T_2 = 4$ using the steady state. We fix $J = 1$, $\gamma _1=0.1$, $\gamma _2=5$, $D_2=5$, ${\bar{n}}_a=2$, ${\bar{n}}_b=0$, $B_1 = 1$ and $B_2 = 2$, and change *D*.

In Fig. 5 using the Eq. (21) contour plots of the quantum coherence is ploted as a function $\gamma _1$ and $\gamma _2$. We find from Fig. 5 which quantum coherence is sensitive to reservoir temperatures. It is important to note under what the condition quantum coherence can be sustain more. We observe that by increasing the interaction rate of any qubit with its environment, the quantum coherence further increases compared to the entanglement (see Figs. 3, 4 and 5 ). As can be seen, the quantum coherence is decreasing in terms of the population of the corresponding equilibrium temperature such as the entanglement and the quantum discord. Also, for constant $\gamma _1$, by increasing the $\gamma _2$, the entanglement becomes more perceptible and the quantum discord the first increases then decreases. But the quantum coherence further increases compared to the entanglement. To compairing the behavior of quantum correlations and quantum work, we draw Fig. 6 and set $D=D_1$. Figure 6 shows the plots of three quantum correlations and the total work, as a function spin-orbit interaction factor *D*. Based on this Figure, *DM* interaction parameter releases the positive work condition compared to export quantum correlations in the *XY* chain under the condition that $D_1 \rightarrow D_2$ in adiabatic process. In the area $D > 2.2$ even when the concurrence is absent, quantum correlation can be exported by way discord, so that positive works happening in our cycle as long as $D\ne 0$. As a result, Fig. 6 shows that the discord of system decreases as the total work done by the system decreases. We can see a clear relation, for this type of engine, between quantum correlations and energetic performances.

## Conclusion

In summary, we proposed a composite system composed of two interactional qubits with spin-orbit interaction. We discussed the work extraction from the engine and its efficiency as well as the role of quantum correlations, characterized by the quantum entanglement, quantum discord and quantum coherence in the thermodynamical processes. The density matrix and quantum correlations are calculated for the state *XY*, and we observe that three quantum correlations decrease by increasing the population of the corresponding equilibrium temperature. We also saw that, in the absence of entanglement in the system, the work done in the system is not necessarily zero, while discord can be a powerful tool for identifying positive work in the system. We also see that with increasing the spin-orbit interaction factor, three quantum correlations and the total work decrease. Therefore, three quantum correlations behavior in terms of the Dzyaloshinskii–Moriya interactions coefficient are the agree on the total work behavior. We have identified for what range of magnetic fields, we can consider this system as a heat engine. Our work show new light on the study of the work extraction from the engine and its efficiency as well as the role of quantum correlations. Interestingly, we have shown that it is possible to make a direct link between the work and the quantum correlations, entanglement, coherence and discord in during the Otto cycle.

## Supplementary Information

Supplementary Information.
