# Supplementary material for: Quantum correlated heat engine in XY chain with Dzyaloshinskii–Moriya interactions
Source: Sci Rep. 2022 Apr 30;12:7081. doi: 10.1038/s41598-022-11146-3 (PMC9056535; doi:10.1038/s41598-022-11146-3)
Supplement: Supplementary file 1 — Supplementary Information. [file 41598_2022_11146_MOESM1_ESM.pdf]

# Quantum correlated heat engine in $XY$ chain with Dzyaloshinskii-Moriya interactions

M. Asadian, S. Ahadpour * & F. Mirmasoudi
Department of Physics, University of Mohaghegh Ardabili 56199-11367, Ardabil, Iran
* Corresponding author. E-mail: Ahadpour@uma.ac.ir

April 3, 2022

## 1 Expressing elements of the X-state density matrix for $XY$ Hamiltonian

In setup considered in the main text, we assume a system consisting of two interacting qubits a and b as the working material a four- level quantum Otto engine, which is described by $XY$ Hamiltonian considering the spin-orbit interaction and magnetic field.

$$H = (J_x\sigma_{x_a}\sigma_{x_b} + J_y\sigma_{y_a}\sigma_{y_b}) + B(\sigma_{z_a} + \sigma_{z_b}) + D(\sigma_{x_a}\sigma_{y_b} - \sigma_{y_a}\sigma_{x_b}), \tag{1}$$

where $\sigma_{[x,y,z]_j}$ denotes the Pauli operators that affect the qubits $j = a, b$, $J_x$ and $J_y$ are the strength of the antiferromagnetic couplings, $B$ is the intensity of the magnetic field, and $D$ is the spin-orbit interaction factor. We set $J_x = J_y = J$ and assume that the dynamics of the working matter density operator $\rho$ have the Markovian property, and can be stated by Lindblad master equation as follows:

$$\dot{\rho} = -[H, \rho] + \sum_i g_i L_{a_i}(\rho). \tag{2}$$

Accordingly, $L_{a_i} = 2a_i\rho^+_{a_i} - \{a^+_i a_i, \rho\}$ and $a_i$ is the jump operator that describes the operation of the baths, and $g_i$ is the dissipation rate associated with the Lindblad term $L_i$ in the above equation. It is assumed that Lindblad operators of $L^j_{\sigma+}$ with coefficient $g^j_+ = \gamma_j\overline{n}_j$, and $L^j_{\sigma-}$ with coefficient $g^j_- = \gamma_j(\overline{n}_j + 1)$ are the jump operators in two general forms of raising operator $\sigma_+ = |1\rangle\langle 0|$ and lowering operator $\sigma_- = |0\rangle\langle 1|$. The coefficients $\gamma_j$ is the interaction rate of each qubit with its surroundings and $\overline{n}_j$ is the population of the corresponding equilibrium temperature which is defined by the following equation:

$$\bar{n}_j = (e^{2B_j/T_j} - 1)^{-1}, \tag{3}$$

where $B_j$ and $T_j$ are the magnetic field and temperature for the bath coupled to qubit $j$, respectively. Under this condition, the density matrix can be expressed as,

$$\rho = \frac{1}{\alpha}\begin{pmatrix} r_{11} & 0 & 0 & 0 \\ 0 & r_{22} & ir_{23} & 0 \\ 0 & -ir_{23} & r_{33} & 0 \\ 0 & 0 & 0 & r_{44} \end{pmatrix}, \tag{4}$$

where $r$'s and $\alpha$ are reported in here. In this section, we provide the analytical formula for the steady state,

$$
\begin{aligned}
\alpha &= (\gamma_1 + 2n_1\gamma_1 + \gamma_2 + 2n_2\gamma_2)^2(4(D^2+J^2) + (1+2n_1)(1+2n_2)\gamma_1\gamma_2),\\
r_{11} &= \frac{4D^2(n_1\gamma_1 + n_2\gamma_2)^2 + 4J^2(n_1\gamma_1 + n_2\gamma_2)^2}{(\gamma_1 + 2n_1\gamma_1 + \gamma_2 + 2n_2\gamma_2)^2(4(D^2+J^2) + (1+2n_1)(1+2n_2)\gamma_1\gamma_2)}\\
&\quad + \frac{n_1 n_2\gamma_1\gamma_2(\gamma_1 + 2n_1\gamma_1 + \gamma_2 + 2n_2\gamma_2)^2}{(\gamma_1 + 2n_1\gamma_1 + \gamma_2 + 2n_2\gamma_2)^2(4(D^2+J^2) + (1+2n_1)(1+2n_2)\gamma_1\gamma_2)},\\
r_{22} &= \frac{4D^2(n_1\gamma_1 + n_2\gamma_2)(\gamma_1 + n_1\gamma_1 + \gamma_2 + n_2\gamma_2)}{(\gamma_1 + 2n_1\gamma_1 + \gamma_2 + 2n_2\gamma_2)^2(4(D^2+J^2) + (1+2n_1)(1+2n_2)\gamma_1\gamma_2)}\\
&\quad + \frac{4J^2(n_1\gamma_1 + n_2\gamma_2)(\gamma_1 + n_1\gamma_1 + \gamma_2 + n_2\gamma_2)}{(\gamma_1 + 2n_1\gamma_1 + \gamma_2 + 2n_2\gamma_2)^2(4(D^2+J^2) + (1+2n_1)(1+2n_2)\gamma_1\gamma_2)}\\
&\quad + \frac{n_1(1+n_2)\gamma_1\gamma_2(\gamma_1 + 2n_1\gamma_1 + \gamma_2 + 2n_2\gamma_2)^2}{(\gamma_1 + 2n_1\gamma_1 + \gamma_2 + 2n_2\gamma_2)^2(4(D^2+J^2) + (1+2n_1)(1+2n_2)\gamma_1\gamma_2)},\\
r_{33} &= \frac{4D^2(n_1\gamma_1 + n_2\gamma_2)(\gamma_1 + n_1\gamma_1 + \gamma_2 + n_2\gamma_2)}{(\gamma_1 + 2n_1\gamma_1 + \gamma_2 + 2n_2\gamma_2)^2(4(D^2+J^2) + (1+2n_1)(1+2n_2)\gamma_1\gamma_2)}\\
&\quad + \frac{4J^2(n_1\gamma_1 + n_2\gamma_2)(\gamma_1 + n_1\gamma_1 + \gamma_2 + n_2\gamma_2)}{(\gamma_1 + 2n_1\gamma_1 + \gamma_2 + 2n_2\gamma_2)^2(4(D^2+J^2) + (1+2n_1)(1+2n_2)\gamma_1\gamma_2)}\\
&\quad + \frac{(1+n_1)n_2\gamma_1\gamma_2(\gamma_1 + 2n_1\gamma_1 + \gamma_2 + 2n_2\gamma_2)^2}{(\gamma_1 + 2n_1\gamma_1 + \gamma_2 + 2n_2\gamma_2)^2(4(D^2+J^2) + (1+2n_1)(1+2n_2)\gamma_1\gamma_2)},\\
r_{44} &= \frac{4D^2(\gamma_1 + n_1\gamma_1 + \gamma_2 + n_2\gamma_2)^2}{(\gamma_1 + 2n_1\gamma_1 + \gamma_2 + 2n_2\gamma_2)^2(4(D^2+J^2) + (1+2n_1)(1+2n_2)\gamma_1\gamma_2)}\\
&\quad + \frac{4J^2(\gamma_1 + n_1\gamma_1 + \gamma_2 + n_2\gamma_2)^2}{(\gamma_1 + 2n_1\gamma_1 + \gamma_2 + 2n_2\gamma_2)^2(4(D^2+J^2) + (1+2n_1)(1+2n_2)\gamma_1\gamma_2)}\\
&\quad + \frac{(1+n_1)(1+n_2)\gamma_1\gamma_2(\gamma_1 + 2n_1\gamma_1 + \gamma_2 + 2n_2\gamma_2)^2}{(\gamma_1 + 2n_1\gamma_1 + \gamma_2 + 2n_2\gamma_2)^2(4(D^2+J^2) + (1+2n_1)(1+2n_2)\gamma_1\gamma_2)},\\
r_{23} &= \frac{2(D - iJ)(n_1 - n_2)\gamma_1\gamma_2}{(\gamma_1 + 2n_1\gamma_1 + \gamma_2 + 2n_2\gamma_2)(4(D^2+J^2) + (1+2n_1)(1+2n_2)\gamma_1\gamma_2)},\\
r_{32} &= \frac{2(D + iJ)(n_1 - n_2)\gamma_1\gamma_2}{(\gamma_1 + 2n_1\gamma_1 + \gamma_2 + 2n_2\gamma_2)(4(D^2+J^2) + (1+2n_1)(1+2n_2)\gamma_1\gamma_2)},\\
r_{41} &= r_{14} = r_{12} = r_{13} = r_{21} = r_{24} = r_{31} = r_{34} = r_{42} = r_{43} = 0.
\end{aligned} \tag{5}
$$
